# Upon the Physical Causes Which Arrest the Progress of Mexican Population

**Published:** 1811-03

**Authors:** 


### *For the Medical and Physical Journal.*

*Upon the Physical Causes which arrest the progress of Mexican population. (Political Essay on the Kingdom of New Spain. By* Alexander de Humboldt. *Translated by* John Black, 1811.*)*

" These causes are the small-pox, the cruel malady called by the Indians *Matlazahuatl*, and especially famine, of which the effects are felt for a long time.

The small-pox, introduced since 1520, appears only to exercise its ravages every seventeen or eighteen years. In the equinoxial regions it has, like the *black vomiting* and several other diseases, its fixed periods, to which it is very regularly subjected. We might say that in these countries the disposition for certain miasmata is only renewed in the natives at long intervals; for though the vessels from Europe frequently introduce the germ of the small-pox, it never becomes epidemical but after very marked intervals; a singular circumstance, which renders the disease so much the more dangerous for adults. The small-pox committed terrible ravages in 1763, and especially in 1779, in which year it carried off in the capital of Mexico alone more than nine thousand persons. Every evening tumbrels passed through the streets to receive the corpses, as at Philadelphia during the yellow fever. A great part of the Mexican youth was cut down that year.

The epidemic of 1797 was less destructive, chiefly owing to the zeal with which inoculation was propagated in the environs of Mexico, and in the bishopric of Mechoachan. In the capital of this bishopric the city of Valladolid, of 6800 individuals inoculated only 170, or 2½ per cent. died; and we must also observe, that several of those who perished were inoculated at a time when they were probably already infected in the natural manner. Fifteen in the hundred died of individuals of all ages, who, without being inoculated, were victims of the natural small-pox. Several individuals, particularly among the clergy, displayed at that period a very praiseworthy patriotism, in arresting the progress of the disease by inoculation. I shall merely mention the names of two enlightened men, M. de Reano, intendant of Guanaxuato, and Don Manuel Abad, penitentiary canon of the cathedral of Valladolid, whose generous and disinterested views were constantly directed towards the public good. There were then inoculated in the kingdom between 50 and 60,000 individuals.

But in the month of January 1804, the vaccine inoculation was even introduced at Mexico through the activity of a respectable citizen, Don Thomas Murphy, who brought several times the virus from North America. This introduction found few obstacles; the cow-pox appeared under the aspect of a very trivial malady; and the small-pox inoculation had already accustomed the Indians to the idea that it might be useful to submit to a temporary evil for the sake of evading a greater evil. If the vaccine inoculation, or even the ordinary inoculation, had been known in the new world in the sixteenth century, several millions of Indians would not have perished victims to the small-pox, and particularly to the absurd treatment by which the disease was rendered so fatal. To this disease the fearful diminution of the number of Indians in California is to be ascribed. The ships of war commissioned to carry the vaccine matter into America and Asia arrived at Vera Cruz shortly after my arrival.

*Don Antonio Valmis*, physician general of this expedition, visited Portorico, Cuba, Mexico, and the Philippine islands; and his stay at Mexico, where nevertheless the cow-pox was known before his arrival, contributed singularly to facilitate the propagation of this salutary preservative. In the principal cities of the kimgdom vaccine committees were formed *(juntas centrales)*, composed of the most enlightened individuals, who, by vaccinating monthly, preserve the miasma from being lost. It is so much the less liable to be lost, as it exists in the country. M. *Valmis* discovered it in the environs of Valladolid, and in the village of Atlisco, near la Puebla, in the udders of the Mexican cows. The commission having fulfilled the beneficent views of the king of Spain, we may indulge a hope that through the influence of the clergy, and especially of the religious missionaries, vaccination will be gradually introduced into the very interior of the country. The voyage of M. Valmis will thus remain for ever memorable in the annals of history. The Indies saw for the first time those vessels, which were formerly freighted only with instruments of carnage and destruction, bearing about the germ of relief and consolation to distressed and suffering humanity.

The arrival of the armed frigates in which M. Valmis made the circuit of the Atlantic and Pacific oceans gave rise on several coasts to one of the most simple, and therefore most affecting, ceremonics. The bishops, military governors, and persons of greatest distinction, repaired to the shore, where they took in their arms the children who were to carry the cow-pox to the indigenous Americans and the Malays of the Philippine islands, and, followed with public acclamations, they laid at the foot of the altar those precious preservative deposits, returning thanks to the Supreme Being for for having been the witnesses of so happy an event. We must have some knowledge of the ravages occasioned by the small-pox in the torrid zone, and especially among a race of men whose physical constitution seems adverse to cutaneous eruptions, in order to feel all the importance of M. Jenner's discovery. It is a much greater blessing for the equinoctial part of the new continent than for the temperate climate of the old.

It may be useful to relate here a fact of some importance, for those who take an interest in the progress of vaccination. It was unknown at Lima till the month of November 1802. At that period the small-pox prevailed on the coast of the South Sea. A merchant vessel, *Santo Domingo de la Calzada*, put into Lima in the passage from Spain to Manilla. An individual had had the good sense to send by this vessel vaccine matter to the Philippine islands. They availed themselves of this opportunity at Lima; and M. Unanue, professor of anatomy, and author of an excellent physiological treatise on the climate of Peru*, vaccinated several individuals by means of the matter brought by the merchant vessel. No pustule appeared; and the virus appeared either altered or too weak. However, M. Unanue having observed that all the vaccinated individuals had a very mild small-pox, employed this variolous matter to render, if possible by the ordinary inoculation, the disease less fatal. He thus perceived in an indirect way the effects of a vaccination supposed to have failed.

It was accidentally discovered in the course of the same epidemic in 1802, that the beneficent effect of vaccination had been long known to the country people among the Peruvian Andes. A negro slave had been inoculated for the small-pox in the house of the Marquis de Valleumbroso who shewed no symptom of the disease. They were going to repeat the inoculation, when the young man told them that he was certain of never having the small-pox, because in milking cows in the Cordillera of the Andes, he had had a sort of cutaneous eruptions, caused, as the Indian herdsmen said, by the contact of certain tubercules sometimes found on the udders of cows. Those who have this eruption, said the negro, never take the small-pox. The Africans, and especially the Indians, display great sagacity in observing the character, habits, and

* This work, which displays an intimate acquaintance with the French and English literature, bears the title of *Observaciones sobre el clima de Lima y sus influencias en los seres organizados en especial el hombre, por el Dr. D. Hipolito Unanue.* Lima, 1806.

 diseases diseases of the animals with which they live. We need not therefore be astonished, that, on the introduction of horned cattle into America, the lower people remarked that the pustules on the udders of cows communicated to the herdsmen a species of benignant small-pox, and that those once infected are secure from the general contagion during the epochs when the disease is epidemical.

The *matlazahuatl*, a disease peculiar to the Indian race, seldom appears more than once in a century. It raged in a particular manner in 1545, 1576, and 1736. It is called a plague by the Spanish authors. As the latest epidemic took place at a time when medicine was not considered a science, even in the capital, we have no exact data as to the *matlazahuatl*. It bears certainly some analogy to the yellow fever or black vomiting; but it never attacks white people, whether Europeans or descendants from the natives. The individuals of the race of Caucasus* do not appear subject to this mortal typhus, while, on the other hand, the yellow fever or black vomiting very seldom attacks the Mexican Indians. The principal site of the *vomito prieto* is the maritime region, of which the climate is excessively warm and humid; but the *matlazahuatl* carries terror and destruction into the very interior of the country, to the central table-land, and the coldest and most arid regions of the kingdom.

Father Torribio a Franciscan, better known by his Mexican name of Motolinia, asserts that the small-pox at its introduction in 1520, by a negro slave of Narvaez, carried off the half of the inhabitants of Mexico. Torquemada advances the hazardous opinion that in the two *matlazahuatl* epidemics of 1545 and 1576, 800,000 Indians died in the former, and 2,000,000 in the latter. But when we reflect on the difficulty with which we can at this day estimate, in the eastern part of Europe, the number of those who fall victims to the plague, we shall very reasonably be inclined to doubt if the viceroys Mendoza and Almanza, governors of a recently conquered country, were able to procure an enumeration of the Indians cut off by the *matlazahuatl*. I do not accuse the two monkish historians of want of veracity; but there is very little probability that their calculation is founded on exact data.

---

* Who are the individuals of the race of Caucasus? The Europeans. So at least we learn from the context, where they are opposed to the Mexican Indians. This involves the theory of the mountains of Asia being the nursery of the old continent. Every one, however, will not so easily be able to understand Europeans by this denomination. Such attempts to elevate the style, at the expence of perspicuity, can never enough be reprobated. *Trans.*

A very interesting problem remains to be resolved. Was the pest, which is said to have desolated from time to time the Atlantic regions of the United States before the arrival of the Europeans, and which the celebrated Rush and his followers look upon as the principle of the yellow fever, identical with the *matlazahuatl* of the Mexican Indians? We may hope that this last disease, should it ever re-appear in New Spain, will be hereafter carefully observed by the physicians.

A third obstacle to the progress of population in New Spain, and perhaps the most cruel of all, is famine. The American Indians, like the inhabitants of Hindostan, are contented with the smallest quantity of aliment on which life can be supported, and increase in number without a proportional increase in the means of subsistence. Naturally indolent, from their fine climate and generally fertile soil, they cultivate as much maize, potatoes, or wheat as is necessary for their own subsistence, or at most for the additional consumption of the adjacent towns and mines. Agriculture, it is true, has made great progress within these last twenty years; but the consumption has also increased in an extraordinary manner from the augmentation of population, and an excessive luxury formerly unknown to the mixed casts, and from the working of a great number of new seams, which require additional men, horses, and mules. Few hands, no doubt, are employed in manufactures in New Spain; but a great number are withdrawn from agriculture from the necessity of transporting on mules goods and the produce of the mines, iron, powder, and mercury, from the coast to the capital, and from thence to the mines along the ridge of the Cordilleras.

Thousands of men and animals pass their lives on the great roads between Vera Cruz and Mexico, Mexico and Acapulco, Oaxaca and Durango, and the cross roads by which provisions are carried to the habitations established in arid and uncultivated regions. This class of inhabitants, called by the economists in their system, sterile and nonproductive, is consequently more numerous in America than might be expected in a country where manufacturing industry is yet so little advanced. The want of proportion between the progress of population and the increase of food from cultivation renews the afflicting spectacle of famine, whenever a great drought or any other local cause has damaged the crop of maize. Scarcity of provisions has always been accompanied in all times and all parts of the globe with epidemical diseases fatal to population*. The want of nourishment in 1784 gave

* This position requires qualification. Dr. Smith has, I believe,

 rise rise to *asthenical* diseases among the most indigent class of people. These accumulated calamities cut off a great number of adults, and a still greater number of children; and it was computed that in the town and mines of Guanaxuato more than 8000 individuals perished. A very remarkable meteorological phenomenon contributed principally to the scarcity: the maize, after an extraordinary drought, was nipt by frost on the night of the 28th of August, and, what is more singular, at an elevation of 1800 metres*. The number of inhabitants carried off by this fatal union of famine and disease throughout the whole surface of the kingdom was estimated at more than 300,000. This number will appear the less astonishing to us when we consider, that even in Europe the population is sometimes diminished by scarcity, more than it is augmented by the excess of births above the deaths for four consecutive years. There perished in Saxony, for example, in 1772, near 66,000 inhabitants, while the excess of births above the deaths was not, *communibus annis*, from 1764 to 1784 more than 17,000†.

---

well remarked that in years of scarcity there are, perhaps, fewer diseases and deaths than usual, from the diminished consumption of spirituous liquors by the common people, one of the most productive sources of disease. The position will undoubtedly, however, hold with regard to a Hindoo or Indian population, who, in years of plenty, have no more than merely supports animal life, and to whom, therefore, any reduction must always prove fatal. *Trans.*

* 5904 feet. *Trans.*

† The translator is afraid that this number of 66,000 includes the whole deaths of Saxony in 1772, in which case the statement that the diminution of population from the famine exceeded the augmentation from the excess of births for four consecutive years will fall to the ground. Every one knows that it is impossible to state exactly the number of deaths from famine in any country, as literally few or none die of famine, but of diseases occasioned by a defective diet, which can never be separated in any bill of mortality from diseases owing to other causes. The nearest approximation, however, is to be found by deducing the average mortality from the increased mortality in any given year of scarcity. I think it extremely probable that M. de Humboldt has not adopted this method. He elsewhere states that the adjacent country of Prussia had, in 1802, on a population of nine millions, 282,109 deaths. If we take Mr. Pinkerton's estimate of the Saxon population, 2,104,000, say, however, 2,000,000, and assume a mortality for it proportionate to that of Prussia, we shall find the number of deaths 62,869. If, supposing then 60,000 the mortality of 1772, and 62,869 the average mortality, the increase by famine in 1772 would only be 3311. This is a much more likely number than the enormous one given by M. de Humboldt; but the fact can easily be ascertained. *Trans.*

The

The effects of famine are common to almost all the equinoxial regions. In the province of New Andalusia in South America I have seen villages whose inhabitants were forced by famine to disperse themselves from time to time in the desarts to pick up a subsistence from the wild plants. In vain the missionaries employ their authority to prevent this dispersion. In the province *de los Pastos*, the Indians when the potatoes fail, which are their principal nourishment, repair sometimes to the most elevated ridge of the Cordillera to subsist on the juice of the achupallas, a plant related to the genus pitcarnia. The Otomacks at Uruana, on the banks of the Orinoco, swallow, during several months, potter's earth, to absorb by this load the gastric juice, and to satisfy, in some sort, the hunger which torments them*. In the islands of the South Sea, in a fertile soil, where nature has lavished all her blessings the inhabitants are frequently driven by famine to devour one another. Under the torrid zone, where a beneficent hand seems every where to have scattered the germ of abundance, man, careless and phlegmatic, experiences periodically a want of nourishment which the industry of more civilized nations banishes from the most sterile regions of the north.

The working of the mines has long been regarded as one of the principal causes of the depopulation of America. It will be difficult to call in question, that at the first epoch of the conquest, and even in the seventeenth century, many Indians perished from the excessive labour to which they were compelled in the mines. They perished without posterity, as thousands of African slaves annually perish in the West Indian plantations from fatigue, defective nourishment, and want of sleep. In Peru, at least in the most southern part, the country is depopulated by the mines, because the barbarous law of the *mita* is yet in existence, which compels the Indians to remove from their homes into distant provinces, where hands are wanted for extracting the subterraneous wealth. But it is not so much the labour as the sudden change of climate, which renders the mita so pernicious to the health of the Indians. This race of men has not the flexibility of organization for which the Europeans are so eminently distinguished. The health of copper-coloured man suffers infinitely when he is transported from a warm to a cold climate, particularly when he is forced to descend from the elevation of the Cordillera into those narrow and humid vallies, where all the miasmata of the neighbouring regions appear to be deposited.

* See my *Tableaux de la Nature*, t. I. P, 62, 191, and 209.

In

In the kingdom of New Spain, at least within the last thirty or forty years, the labour of the mines is free; and there remains no trace of the *mita*, though a justly celebrated author* has advanced the contrary. No where does the lower people enjoy in greater security the fruit of their labours than in the mines of Mexico; no law forces the Indian to choose this species of labour, or to prefer one mine to another; and when he is displeased with the proprietor of the mine, he may offer his services to another master who may pay, perhaps, more regularly. These unquestionable facts are very little known in Europe. The number of persons employed in subterraneous operations, who are divided into several classes (*Barenadores*, *Faeneros*, *Tenateros*, *Bareteros*), does not exceed in the whole kingdom of New Spain 28 or 30,000. Hence there is not more than $\frac{1}{200}$ of the whole population immediately employed in the mines.

The mortality among the miners of Mexico is not much greater than what is observed among the other classes. We may easily be convinced of this by examining the bills of mortality in the different parishes of Guanaxuato and Zacatecas. This is a phenomenon, so much the more remarkable, as the miner in several of these mines is exposed to a temperature 6 deg. above the mean temperatures of Jamaica and Pondicherry.† I found the centrigrade thermometer at 34 deg. ‡ at the bottom of the mine of Valenciana (*en los planes*), a perpendicular depth of 513 metres,§ while at the mouth of the pit in the open air, the same thermometer sinks in winter to 4 deg. or 5 deg. || above 0. The Mexican miner is consequently exposed to a change of temperature of more than 30 deg. **. But this enormous heat of the Valenciana mine is not the effect of a great number of men and lights collected into a small space; it is much more owing to local and geological causes which we shall afterwards examine.

It is curious to observe how the Mestizoes and Indians employed in carrying minerals on their back, who go by the name of *Tenateros*, remain continually loaded for six hours with a weight of from 225 to 350 pounds, and constantly exposed to a very high temperature, ascending eight or ten times successively, without intermission, stairs of 1800 steps.

---

* Robertson's History of America, vol. ii. p. 378.
† Neariy 11 deg. of Fahrenheit. *Trans.*
‡ 93 deg. of Fahrenheit. *Trans.* § 1681 feet. *Trans.*
|| 39 deg. or 41 deg. of Fahrenheit. *Trans.*
** 54 deg. of Fahrenheit. *Trans.*

The

The appearance of these robust and laborious men would have operated a change in the opinions of the Raynals and Pauws, and a number of other authors, however estimable in other respects, who have been pleased to declaim against the degeneracy of our species in the torrid zone. In the Mexican mines, children *(enfans)* of seventeen years of age* are able to carry masses of stone of a hundred pounds weight. This occupation of Tenateros is accounted unhealthy, if they enter more than three times a week into the mines. But the labour which ruins most rapidly the robustest constitutions is that of the *Barenadores*, who blow up the rock with powder. These men rarely pass the age of 35, if from a thirst of gain they continue their severe labour for the whole week. They generally pass no more than five or six years at this occupation, and then betake themselves to other employments less injurious to health.

The art of mining is daily improving, and the pupils of the school of mines at Mexico gradually diffuse correct notions respecting the circulation of air in pits and galleries. Machines are beginning to be introduced in place of the old method of carrying minerals and water on men's backs up stairs of a rapid ascent. In proportion as the mines of New Spain resemble more and more those of Freiberg, Clausthal, and Schemnitz, the miner's health will be less injured by the influence of the *Mofettes**, and the excessively prolonged efforts of muscular motion.

From five to six thousand persons are employed in the amalgamation of the minerals, or the preparatory labour. A great number of these individuals pass their lives in walking barefooted over heaps of brayed metal, moistened and mixed with muriate of soda, sulphate of iron, and oxide of mercury, by the contact of the atmospheric air and the solar rays. It is a remarkable phenomenon to see these men enjoy the most perfect health. The physicians who practice in places where there are mines unanimously assert, that the nervous affections, which might be attributed to the effect of an absorption of oxid of mercury, very rarely occur. At Guanaxuato part of

* I should be inclined to think that the author meant to say *enfans de sept à dix ans*, instead of *enfans de dix sept ans*, for *enfant*, it is believed; can hardly be applied with propriety to a youth of 17; and, if a full-grown man could ascend eight or ten times, without intermission, 1800 steps of a stair with 350 pounds, it certainly could not add to the evidence of the strength of this race to say, that a young man of 17 could carry little more than the fourth part of that weight. *Trans.*

† The translator professes his ignorance of the meaning of this word.

the the inhabitants drink the very water in which the amalgamation has been purified *(aqua de lavaderos)* without feeling any injury from it. This fact has often struck Europeans not intimately acquainted with the principles of chemistry. The water is at first of a greyish-blue colour, and contains in suspension black oxid of mercury, and small globules of native mercury and amalgamation of silver. This metallic mixture gradually precipitates, and the water becomes limpid. It can neither dissolve the oxid of mercury nor the muriate of mercury, which is one of the most insoluble salts which we know. The mules are very fond of this water, because it contains a little muriate of soda in dissolution.

In speaking of the progress of the Mexican population, and of the causes which retard that progress, I have neither mentioned the arrival of new European colonists, nor the mortality occasioned by the *black vomiting*. We shall discuss these subjects in the sequel. It is sufficient to observe here, that the *vomito prieto* is a scourge which is never felt but on the coast, and which does not, throughout the whole kingdom, carry off annually more than from two to three thousand individuals. As to Europe, it does not send more than 800 to Mexico. Political writers have always exaggerated what they call the depopulation of the old continent by the new. M. Page*, for example, asserts in his work on the commerce of St. Domingo, that the emigrations from Europe supply annually more than 100,000 individuals to the United States. This estimate is twenty times higher than the truth; for, in 1784 and 1792, when the United States received the greatest number of European colonists, their number did not exceed 5000†. The progress of population in Mexico and North America is solely derived from an increase of internal prosperity.

